# Autogenous paneled femoral vein grafts for mycotic thoracic aortic aneurysms

**DOI:** 10.1016/j.xjtc.2025.07.008

**Published:** 2025-07-19

**Authors:** Ningzhi Gu, Eimaan Shergill, D. Kirk Lawlor, Michael Janusz, Jong Moo Kim, Joel Price, Jason Faulds

**Affiliations:** a Division of Vascular Surgery, University of British Columbia, Vancouver, British Columbia, Canada; b Faculty of Medicine, University of British Columbia, Vancouver, British Columbia, Canada; c Division of Cardiac Surgery, Department of Surgery, University of British Columbia, Vancouver, British Columbia, Canada

**Keywords:** mycotic aneurysm, thoracic aortic aneurysm, femoral vein graft

## Abstract

**Objective:**

Mycotic thoracic aortic aneurysms (MTAAs) are rare. The most common management has been aortic resection, wide debridement, and in-line reconstruction using prosthetic grafts. We have used autogenous femoral vein (FV) for the repair of all MTAA in our institution since 2017. This is the initial description of this technique, and the first comparative study of autogenous vein compared with prosthetic for MTAAs.

**Methods:**

This is a single-center retrospective cohort study of all patients with MTAA who underwent operative repair. Patients were assigned to the FV or prosthetic grafts cohorts. Perioperative and long-term outcomes were collected. Univariate logistic regression models were fitted to quantify the strength of differences between the cohorts.

**Results:**

Nineteen patients were included. The first 9 consecutive patients had prosthetic grafts, whereas the 10 subsequent patients were treated with FV grafts. Patients in the FV cohort were more likely to have positive intraoperative cultures (90% vs 33.3%; *P* = .02), receive intraoperative transfusions (10 vs 8 units; *P* = .08), and have a longer operation (629 vs 500 minutes; *P* = .07). There was a trend toward improved in-hospital (0 vs 33%; *P* = .09) and long-term mortality (10% vs 55.6%; *P* = .57) in the FV cohort. Patients in the FV cohort were more likely to be discharged home (90% vs 44.4%; *P* = .05).

**Conclusions:**

Paneled autogenous FV repair is a durable and safe treatment for patients with MTAA. There were no in-hospital deaths in our series and there have been no long-term complications related to the vein graft repair.

**Figure:**
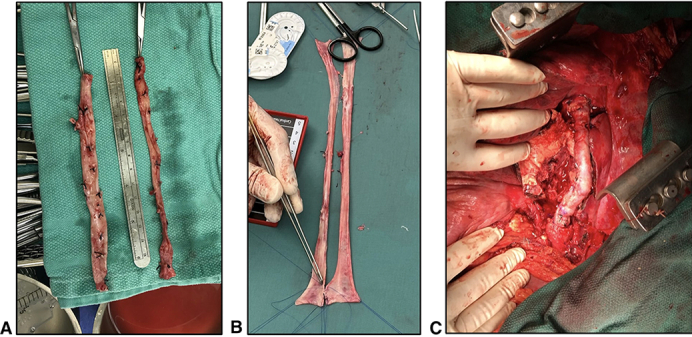
A, Harvested femoral veins. B, Creation of paneled vein graft. C, Paneled vein graft in situ.

**Central Message**

The use of an autogenous vein graft for repair of mycotic thoracic aneurysms is safe. It may be associated with improved mortality and discharge but is challenging and requires longer operative time.

**Perspective**

The standard of care for mycotic thoracoabdominal aneurysms is wide debridement followed by reconstruction with a prosthetic conduit. The outcomes of this are varied; there is a substantial reinfection risk. Endovascular repair has been described but is reserved for high-risk patients. In this study, we describe our experience of mycotic thoracoabdominal aneurysm repair with femoral vein grafts.

Mycotic thoracic aortic aneurysms (MTAAs) are a rare clinical entity.^1^ Without an aggressive surgical approach, the natural history of this condition is poor. The standard of care is a wide surgical debridement, followed by in-line open reconstruction with a prosthetic conduit. Thoracic endovascular aortic repair (TEVAR) has been advocated as a treatment option, but is often reserved for selected high-risk patients or as a bridge to definitive surgery.^2^ The results of prosthetic conduits in the literature are heterogeneous, with varying long-term outcomes reported.^2^^,^^3^ In our experience, we have noted prohibitive mortality and reinfection risks with prosthetic conduits despite wide surgical debridement and soft tissue coverage with either an omental pedicle or intercostal muscle flap. The use of an autogenous femoral vein (FV) as a conduit for infections of the infrarenal aorta is well described, initially introduced by Clagett^4^ as the neoaortoiliac system procedure for abdominal aortic infections. At our institution, we have implemented a similar strategy for repair of MTAAs. We surmise that the persistent reinfection risk in patients undergoing open reconstruction for MTAA can be further reduced by using an autogenous conduit, with minimal additional morbidity. The purpose of this study was to provide an initial description of this technique, as well as to offer a comparative study of autogenous FV compared with prosthetic conduits for surgical treatment of MTAAs.

## Methods

A retrospective review of all patients undergoing open descending and thoracoabdominal aortic aneurysm repair at the University of British Columbia between 2005 and 2023 was conducted. The University of British Columbia Research Ethics Board approved the study protocol and publication of data (H23-03098; February 26, 2024). Patient written consent for the publication of the study data was waived by the research ethics board. Patients with MTAA were identified and selected for further study based on a constellation of clinical features, which included sepsis or bacteremia, radiographic findings, or a clinical picture of secondary aortitis such as aortoesophageal or aortobronchial fistula. Due to a small number of patients with mycotic aneurysms, we elected to include both descending and those requiring thoracoabdominal aortic exposure in the study. Following review of the operative report, patients were assigned to the FV or prosthetic graft (PG) cohorts depending on the conduit used in the aortic reconstruction. Perioperative and long-term outcomes were collected through chart review. Continuous variables such as age and operative time were summarized using medians (interquartile range) and tested for group differences with the Wilcoxon rank-sum test. Categorical baseline factors were summarized using frequency and percentage by cohort using Fisher exact test. Univariate logistic regression models were fitted to quantify the strength of differences between the cohorts.

### Patients

All patients with MTAA are treated collaboratively by cardiac and vascular surgeons in our complex thoracic aortic program. We favor an aggressive surgical approach for patients with MTAA and we rarely defer surgery for nonoperative attempts at managing aortic infections such as drains or prolonged periods of antibiotic therapy. For cases with obvious esophageal or bronchial involvement, thoracic surgeons are involved for esophageal or pulmonary resection.

### Technique

Our standard thoracoabdominal aortic spinal cord protection adjuncts include neurophysiologic monitoring, mild hypothermia to 34 °C, cerebrospinal fluid (CSF) drainage, and distal aortic perfusion using left atrial to femoral bypass or venoarterial extracorporeal membrane oxygenation. In cases of circulatory arrest, CSF drainage and neurophysiologic monitoring are not used. In cases where motor evoked potentials drop below 50% of baseline, spinal cord rescue maneuvers are initiated, which include blood pressure augmentation, blood transfusion, and increased CSF drainage.

**Figure 1:**
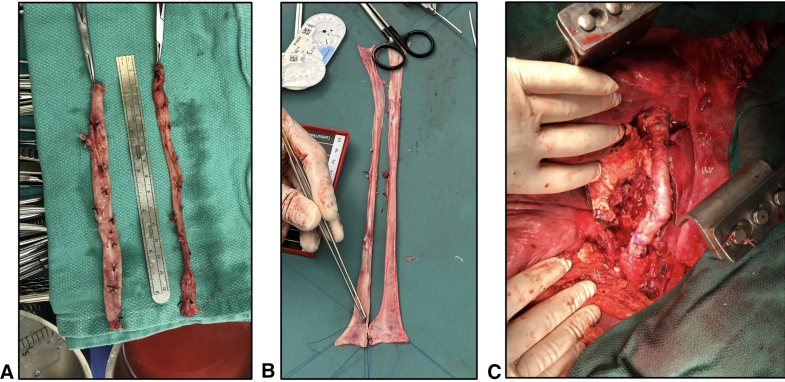
Creation and implantation of a panelled femoral vein graft. A, Harvested femoral veins. B, Creation of paneled vein graft. C, Paneled vein graft in situ.

Following intubation with an endobronchial tube, the operation commences in the supine position. Working in 2 teams, both superficial FVs are procured simultaneously from the proximal popliteal fossa to the level of the confluence with the deep FV. The deep FV is preserved to prevent venous hypertension in the limb. To facilitate wound healing, skip incisions are used when possible. The wounds are closed except for the left groin wound, which is temporarily closed with clips to allow easy access to the femoral artery for bypass cannulation once the patient is repositioned. Following closure of the leg wounds, the procured superficial FVs are opened to form 2 long panels, which are sewn together using 4-0 polypropylene suture over a large diameter chest tube to form a single, large diameter conduit (Figure 1).

The patient is then transferred to the right lateral decubitus position and draped. A standard thoracic or thoracoabdominal aortic exposure is performed depending on the extent of mycotic aneurysm extension. In cases of rupture, cardiopulmonary bypass is initiated before opening the chest.

When proximal aortic clamping cannot be safely completed due to dense scar tissue from previous surgical dissection or in cases of infected TEVARs extending into the distal arch, circulatory arrest is used. In these cases, we are diligent to avoid air entrapment due to the inherent challenges of de-airing the heart through the left chest exposure.

Wide surgical debridement of all infected aortic tissue with complete resection of all foreign material is critical. In cases of aorto-esophageal fistula, esophagectomy is completed with the creation of a cervical esophagostomy to mitigate the risk of esophageal leak. In all cases, esophageal reconstruction is completed within 1 year of the aortic repair.

The proximal anastomosis is completed with 3-0 polypropylene suture in the standard fashion. Vein-pledgeted sutures can be used to reinforce the proximal anastomosis, although this is infrequently required. The graft is oriented such that the paneled suture lines are easily accessible for repair sutures if needed. Instead of disrupting the paneled graft suture lines by cutting the paneled femoral vein graft, the vein is rolled up to the appropriate length and sewn without cutting the graft.

In nonmycotic thoracic and thoracoabdominal aortic repair, our group is aggressive in reimplanting intercostal arteries to reduce immediate and delayed paraplegia. In cases of MTAA, we did not reimplant any intercostals. In cases requiring an extent 1 thoracoabdominal repair, we beveled our distal aortic anastomosis to include the celiac artery. We did not directly reimplant any visceral vessels.

We used adjunctive omental flaps in all patients who received a PG. A tongue of omentum was mobilized from the transverse colon and brought up through a small hole in the diaphragm to cover the graft. Omental flaps were not used in the FV cohort.

As with all thoracoabdominal aortic repairs, meticulous attention to hemostasis is required. We did not place any surgical drains. In all cases, the chest is closed in the operating room and not left open or packed. The patient is then managed in the surgical intensive care unit in the standard manner. We followed our grafts with computed tomography angiography, the first obtained just before discharge and yearly thereafter.

Parenteral antibiotics, targeted if possible toward the isolated organism, were continued for a minimum of 6 weeks postoperatively. Antibiotics were then discontinued in the FV cohort. All patients in the PG cohort were maintained on suppressive oral antibiotics indefinitely.

## Results

Nineteen patients underwent operative repair for MTAA during the study duration period. No patients with MTAA were excluded from the analysis. The first 9 consecutive patients (2005-2017) were repaired using PG and the next 10 consecutive patients (2017-2023) were treated with paneled FV grafts. There were no significant differences in baseline demographic features between cohorts (Table 1). One patient in each group was treated for rupture. Primary mycotic aneurysms were rare and not significantly different between cohorts. TEVAR removal occurred in 5 patients in the FV cohort and in 2 patients in the PG cohort. Thoracoabdominal exposure was required in 5 patients in the FV cohort, all of which were extent I, and 1 patient in the PG cohort, also extent I. Three patients in the FV cohort and 4 in the PG cohort required esophagectomy; amongst the esophagectomy patients, those who survived the initial operation all were treated with delayed esophageal reconstruction without complication.

**Table 1:** Comparison of baseline demographic features between cohorts

Variable	Polyethylene terephthalate cohort (n = 9)	Vein cohort (n = 10)	*P* value∗
Median age at procedure (y)	67.0 (65.0, 70.0)	58.5 (41.0, 63.0)	.01†
Male sex	4 (44.4)	8 (80.0)	.17
Coronary artery disease	2 (22.2)	1 (10.0)	.58
Previous thoracic aortic surgery	6 (66.7)	7 (70.0)	.99
Hypertension	4 (44.4)	5 (50.0)	.99
Dyslipidemia	2 (22.2)	4 (40.0)	.63
Diabetes	1 (11.1)	1 (10.0)	.99
Former or current smoker	5 (55.6)	5 (50.0)	.99
Emergency or rupture	1 (11.1)	1 (10.0)	.99
Coarctation repair	0	2 (20.0)	.47
TEVAR removal	2 (22.0)	5 (50.0)	.37
Previous open repair traumatic injury	0	2 (20.0)	.47
Primary mycotic aneurysm	2 (22.0)	4 (30.0)	.63
Aorto-esophageal fistula	4 (44.4)	3 (30.0)	.65
Aorto-bronchial fistula	0	1 (10.0)	.99

Values are presented as median (Q1, Q3) or n (%). *TEVAR*, Thoracic endovascular aortic repair.

∗ All testing based on Fisher exact test unless otherwise noted.

† Wilcoxon rank sum test.

In 4 of 10 patients in the FV cohort, 1 FV was procured. The remaining patients required both FVs to be taken due to the anticipated length of graft required. We did not reimplant intercostals in any of the patients treated for MTAA. There was a single case of paraplegia in the PG cohort in a patient who subsequently died. Patients in the FV cohort were more likely to have positive intraoperative cultures (90% vs 33.3%; *P* = .02) compared with those in the PG cohort. Species isolated included *Salmonella* in 2 patients, *Staphylococcus aureus* in 2 patients, *Bacteroides* species in 2 patients, *Klebsiella* in 1 patient, and *Scopulariopsis brevicaulis* in 1 patient. In the PG cohort, *Proteus, Bacteroides, Klebsiella*, and *Salmonella* were isolated.

One patient in the FV cohort required reoperation for bleeding from the proximal and distal anastomoses, which were suture-repaired. He then had a blowout of the distal segment of his vein graft, presumably from infection. His vein graft was removed and exchanged for a rifampin-soaked polyethylene terephthalate graft. The patient survived this second operation with no signs of reinfection at 1 year follow-up. One patient in the PG cohort was found to have a contained rupture of his proximal anastomosis 3 weeks following his index procedure, presumably from extension of his previous infection. He was ultimately palliated.

Overall, patients in the FV cohort received more intraoperative transfusions (10 vs 8 U; *P* = .08) and had a longer operating room time (629 vs 500 minutes; *P* = .07) (Table 2). There was a trend toward improved in-hospital mortality (0 vs 33%; *P* = .09) and long-term mortality (10% vs 55.6%; *P* = .57) in the FV cohort (Table 3). Patients in the FV cohort were more likely to be discharged to home (90% vs 44.4%; *P* = .05) (Table 3). There was no aneurysmal degeneration of any of our vein grafts at a mean follow-up of 40 months (Table 4). There were no cases of venous hypertension in the FV cohort, and none of these patients experienced wound complications related to vein harvesting.

**Table 2:** Comparison of intraoperative outcomes between cohorts

Variable	Polyethylene terephthalate cohort (n = 9)	Vein cohort (n = 10)	*P* value∗
Thoracoabdominal exposure	1 (11.1)	5 (50.0)	.14
Esophagectomy	4 (44.4)	3 (30.0)	.65
Circulatory arrest	2 (22.2)	2 (20.0)	.99
No growth from specimen	6 (66.7)	1 (10.0)	.02
Highest lactate level (mmol/L)	3.1 (2.1, 3.5)	2.4 (2.1, 2.7)	.71†
Lowest pH	7.28 (7.23, 7.34)	7.26 (7.21, 7.36)	.65†
Lowest glomerular filtration rate	37.0 (22.0, 62.0)	60.5 (44.0, 99.0)	.25†
Intraoperative red blood cells transfused (U)	8.0 (4.0, 10.0)	10.0 (6.0, 12.0)	.08†
Total red blood cells transfused (U)	8.0 (5.0, 12.0)	11.0 (9.0, 14.0)	.13†
Operative time (min)	500.0 (378.0, 532.0)	629.0 (497.0, 897.0)	.07†

Values are presented as median (Q1, Q3) or n (%).

∗ All testing based on Fisher exact test unless otherwise noted.

† Wilcoxon rank sum test.

**Table 3:** Postoperative and long-term outcomes between cohorts

Variable	Polyethylene terephthalate cohort (n = 9)	Vein cohort (n = 10)	*P* value∗
In-hospital mortality	3 (33.3)	0	.09
Long-term mortality	5 (55.6)	1 (10.0)	.05
Paraplegia	1 (11.1)	0	.47
Stroke	0	1 (10.0)	.99
Myocardial infarction	0	0	–
Days on ventilator	7.0 (2.0, 10.0)	2.5 (1.0, 4.0)	.13†
Tracheostomy	2 (22.2)	0	.21
Discharged to home	4 (44.4)	9 (90.0)	.05
Length of stay (d)	23.0 (14.0, 33.0)	29.0 (19.0, 33.0)	.71†
Unplanned reoperation	0	1 (10.0)	.99

Values are presented as median (Q1, Q3) or n (%).

∗ All testing based on Fisher exact test unless otherwise noted.

† Wilcoxon rank sum test.

**Table 4:** Long-term outcomes of femoral vein grafts

Duration of follow-up (mo)	Maximum vein diameter at original CT (mm)	Maximum vein diameter at original CT (mm)
7	23	23
12	22.5	23
16	19	19
24	11	12
40	30	29
42	17	23.5
63	18	16.7
68	17.8	19
84	16.1	16

*CT*, Computed tomography.

## Discussion

MTAAs are a rare but highly lethal variant of aortic aneurysms.^2^ Bacteremia in the presence of an existing aneurysm, erosion into surrounding structures, contamination of a TEVAR and penetrating trauma are all reported causes of MTAA. These infections remain quite rare, representing <1% of all aortic cases in most aortic centres,^5^ although this varies by region.^6^ The underlying pathophysiology and significantly worse natural history are distinct to that of degenerative aneurysms, necessitating an aggressive clinical approach.

This single-center review demonstrates the safety and efficacy of using autogenous FV for the repair of MTAAs and thoracoabdominal aortic aneurysms. Despite the complexity of these patients and the repair, the outcomes seen in our FV cohort are excellent, with no perioperative mortality and 90% survival at a mean follow-up of 40 months. When compared with the polyethylene terephthalate cohort, there is a trend toward longer operative time and need for blood transfusion. This is offset by a trend toward decreased perioperative and long-term mortality, as well as days intubated, and increased rate of discharge to home. The durability of autogenous conduit is confirmed not only in these outcomes, but also in long-term follow-up. There has been no evidence of degeneration of the implanted vein grafts. Although many of the differences found between the 2 cohorts are trends, these findings support our institutional approach of using autogenous vein as the conduit of choice in patients with MTAA.

Patients with mycotic aneurysms of the descending thoracic aorta likely have improved survival compared with those with mycotic thoracoabdominal aortic disease. Certainly, the surgical insult of an open descending repair is much less than that required in thoracoabdominal aortic repair. Due to the small number of patients with mycotic aneurysms in our series, we elected to group these patients together. Larger series, or systematic review, may provide better insight into the differences in outcome between patients with mycotic descending and thoracoabdominal aortic disease.

Cryopreserved allografts have emerged as an alternative conduit choice in primary and secondary aortic infections. This option spares the morbidity and time associated with vein harvest while theoretically exhibiting a reduced infection rate that is conferred by human tissue. At our institution, we are aggressive in using the superficial FV as a conduit in vascular reconstruction and likely due to our technique of preserving the deep FV, venous complications are exceedingly rare. Indeed, at follow-up there were no symptoms of venous hypertension in our series. In an impressive series, Corvera and colleagues^7^ reviewed 50 consecutive patients who underwent in-line repair of an infected thoracic or thoracoabdominal aortic graft or mycotic aneurysm with cryopreserved allografts. Most of their cohort had infected grafts (66%). Their perioperative mortality was 8%, with a reasonable subsequent 64% 5-year overall survival. The graft infection rate was 12%, as demonstrated by development of pseudoaneurysms (8%) or aortoesophageal fistulas (4%). The mortality associated with graft infections was 4%. Graft infection rates vary quite substantially, even between large studies. Zhou and colleagues^8^ demonstrated, in 4 major American aortic centers, no graft infection in a cohort of 42 patients at a mean follow-up of 1 year. A French study cohort had a graft infection rate of 2.8% in their cohort of 70 patients at a median follow-up of 26.5 months.^9^ Our own institutional experience with cryopreserved grafts, although mostly confined to the abdominal aorta, is congruent with these findings because there is a low but definite risk of graft infection requiring reintervention. Another concern related to the use of cryopreserved allografts is the risk of graft rupture. A meta-analysis on the use of cryopreserved allografts for aortoiliac infection by Antonopoulos and colleagues^10^ reported complication rates of 3% to 6% for anastomotic rupture, allograft degeneration, and pseudoaneurysm formation. Vogt and colleagues^11^ reported 8 (16%) allograft-related complications out of a cohort of 49 patients, which included intraoperative rupture, early postoperative rupture, allograft-enteric fistulas originating from ruptured side branches, anastomotic stricture, and late anastomotic failure. In another study of 55 patients who underwent infected abdominal aortic aneurysm replacement with cryopreserved allograft, 5 (9%) had persistent infection with perianastomotic hemorrhage and 1 patient (2%) had pseudoaneurysm formation.^12^ These findings raise concerns regarding the long-term viability of cryopreserved allografts for infected aortic repair.

Generally, the placement of prosthetic material in an infected field violates a basic surgical principle. Despite this, many institutions advocate for the use of TEVAR as an initial treatment option in MTAA. Certainly, this is a less morbid option compared with open repair. In a review of Swedish registry data from 2000 to 2016, only 4% of mycotic aneurysms were treated with wide debridement and open repair.^13^ They did demonstrate a reasonable survival of 92% at 30 days and 71% at 5 years, but up to a fifth of their cohort developed graft related complication (sepsis and graft infection). Most of these complications were fatal. A subsequent review of mycotic aortic aneurysms treated with stent grafting at multiple European centers demonstrated similar results, with a perioperative survival rate of 91% and a reinfection rate of 27% at a mean follow up of 35 weeks.^14^ This review demonstrated a 5-year survival of only 55%, with 19% of the total cohort dying from a graft-related infectious complication. We have minimal experience using endovascular grafts in MTAA but have found they increase the extent of the definitive operation, particularly when used in the proximal descending thoracic aorta and distal arch. In these cases, the likelihood of circulatory arrest being required to resect the endovascular graft is high.

The limitations of this study are in keeping with those of small, single-center retrospective reviews. The retrospective nature of the study may introduce bias. As previously mentioned, the small size of our cohort makes it difficult to mitigate heterogeneity in our 2 cohorts and draw definitive conclusions. The FV cohort, when compared with the polyethylene terephthalate graft cohort, is significantly younger. In addition, all FV repairs were performed after all polyethylene terephthalate graft repairs, introducing the possibility of more experience and better critical care. These factors may explain some the differences in outcomes between the cohorts. Overall, it may also be difficult to generalize these findings because significant experience with open thoracic and thoracoabdominal aortic disease is required to offer successful treatment to those with mycotic thoracic aortic pathology.

## Conclusions

In summary, the use of autogenous FV for repair of mycotic thoracic and thoracoabdominal aortic aneurysms is safe when compared with traditional repair with prosthetic conduits. The procedure is more technically demanding and is associated with increased operative time and blood loss but demonstrates a trend toward improved survival and durable long-term results. Further studies or a clinical registry, as suggested by previous authors, would be helpful in determining the generalizability of this technique and its benefit over traditional prosthetic repair.

## Conflict of Interest Statement

The authors reported no conflicts of interest.

The *Journal* policy requires editors and reviewers to disclose conflicts of interest and to decline handling or reviewing manuscripts for which they may have a conflict of interest. The editors and reviewers of this article have no conflicts of interest.
